# Post-Learning Sleep Transiently Boosts Context Specific Operant Extinction Memory

**DOI:** 10.3389/fnbeh.2017.00074

**Published:** 2017-04-26

**Authors:** Margarita Borquez, María P. Contreras, Ennio Vivaldi, Jan Born, Marion Inostroza

**Affiliations:** ^1^Departamento de Psicología, Universidad de ChileSantiago, Chile; ^2^Institute of Medical Psychology and Behavioral Neurobiology, University of TübingenTübingen, Germany; ^3^Instituto de Ciencias Biomédicas, Universidad de ChileSantiago, Chile; ^4^German Center for Diabetes Research (DZD), Institute for Diabetes Research and Metabolic Diseases of the Helmholtz Center Munich at the University of Tübingen (IDM)Tübingen, Germany; ^5^Centre for Integrative Neuroscience, University of TübingenTübingen, Germany

**Keywords:** sleep, operant extinction, recent memory, remote memory, generalization, context

## Abstract

Operant extinction is learning to supress a previously rewarded behavior. It is known to be strongly associated with the specific context in which it was acquired, which limits the therapeutic use of operant extinction in behavioral treatments, e.g., of addiction. We examined whether sleep influences contextual memory of operant extinction over time, using two different recall tests (Recent and Remote). Rats were trained in an operant conditioning task (lever press) in context A, then underwent extinction training in context B, followed by a 3-h retention period that contained either spontaneous morning sleep, morning sleep deprivation, or spontaneous evening wakefulness. A recall test was performed either immediately after the 3-h experimental retention period (Recent recall) or after 48 h (Remote), in the extinction context B and in a novel context C. The two main findings were: (i) at the Recent recall test, sleep in comparison with sleep deprivation and spontaneous wakefulness enhanced extinction memory but, only in the extinction context B; (ii) at the Remote recall, extinction performance after sleep was enhanced in both contexts B and C to an extent comparable to levels at Recent recall in context B. Interestingly, extinction performance at Remote recall was also improved in the sleep deprivation groups in both contexts, with no difference to performance in the sleep group. Our results suggest that 3 h of post-learning sleep transiently facilitate the context specificity of operant extinction at a Recent recall. However, the improvement and contextual generalization of operant extinction memory observed in the long-term, i.e., after 48 h, does not require immediate post-learning sleep.

## Introduction

Sleep has been identified as a state that optimizes the consolidation of newly acquired memory (Born et al., 2006; Ribeiro, 2012). Memory consolidation refers to the progressive post-acquisition stabilization of long-term memory (Dudai, 2004). According to the active systems consolidation view, sleep supports the formation of long-term memories through the repeated reactivation of newly encoded representations occurring during sleep (Dudai, 2004; Diekelmann and Born, 2010). Such reactivations mediate the gradual redistribution of the representation from networks serving as initial store to networks serving as long-term store. Importantly, the redistribution process is thought to go along with a transformation of the representation into more generalized memory that becomes decontextualized, i.e., independent from the context in which it was originally learned (Rasch and Born, 2013).

In operant conditioning the individual associates an (operant) behavior with its rewarding or aversive consequences. Extinction refers to the fact that the operant behavior diminishes when the consequences do not occur on repeated occasions (Skinner, 1953). Extinction is considered not to erase the original learning, but rather to represent a new learning to suppress the original behavior (Bouton and Ricker, 1994). It is particularly sensitive to the context in which extinction is acquired; typically, the originally learnt operant behavior reappears (renewal) once the animal is removed from the context in which extinction was originally acquired (Bouton and Swartzentruber, 1991; Nakajima et al., 2000; Rescorla, 2008; Bouton et al., 2011; Polack et al., 2012; Todd et al., 2012; Todd, 2013). Indeed, context specificity of extinction is the most important limiting factor in the clinical application of extinction-based therapies, e.g., in drug addiction, with frequent relapses once the patient leaves the therapeutic setting.

Based on the body of literature showing that sleep supports the formation of generalized context-independent memory representations, we explored whether sleep can make extinction memories less specific to the context in which they were originally acquired. Previous studies on this matter do not provide a conclusive picture. Sleep deprivation impaired extinction of conditioned fear (Hunter, 2015) and extinction (but not acquisition) of an appetitive behavior in bees (Hussaini et al., 2009). In humans, sleep supported the generalization of an extinction memory from an extinguished conditioned stimulus (colored lamps shown on a screen) to a similar stimulus that was not previously used during extinction (Pace-Schott et al., 2009). Also, deprivation of sleep or of REM sleep impaired context specific extinction in a cued fear conditioning paradigm (Silvestri, 2005; Pace-Schott et al., 2012; Melo and Ehrlich, 2016). Whereas these studies altogether support the view that sleep enhances *cue generalization* in the extinction of a classically conditioned aversive response, and also underlying the clinical relevance of this effect, it is less clear whether sleep after extinction training similarly supports the *context generalization* of an extinguished response. There is one study that provided first hints at a possible positive effect of sleep on the context generalization of extinction learning, using a classically conditioned fear response (Kleim et al., 2014). However, so far no study tested the effects of sleep after extinction training using an operantly conditioned response, despite the obvious clinical relevance of this issue, e.g., for treating addictive behavior. Here, we compared the effects of a 3-h period of sleep (vs. sleep deprivation or spontaneous wakefulness) on the memory for the extinction of an operant behavior in different groups of rats. Extinction memory was tested in the context where extinction was originally learned and in a novel context in order to assess the generalization of the memory across different contexts, with context order balanced across individuals. To address the fact that decontextualized memory often emerges only with some delay after the original learning (Winocur et al., 2007), we tested recall of the extinguished response immediately after the 3-h retention period and, in different groups, after a 48-h delay period.

## Materials and Methods

### Animals

Seventy-six adult male Sprague-Dawley rats (obtained from the breeding colony vivarium at the Facultad de Medicina of the Universidad de Chile; ~3-months old; 250–350 g) were used for the experiments. Rats were housed individually, kept in a 12-h light/12-h dark cycle with lights switched on at 07:00 a.m. Water was freely available throughout the experiment. All experimental procedures were approved by the animal welfare committee of the Universidad de Chile (CBA# 0797 Facultad de Medicina, Universidad de Chile, FMUCH).

### Design and Procedure

**Figure 1:**
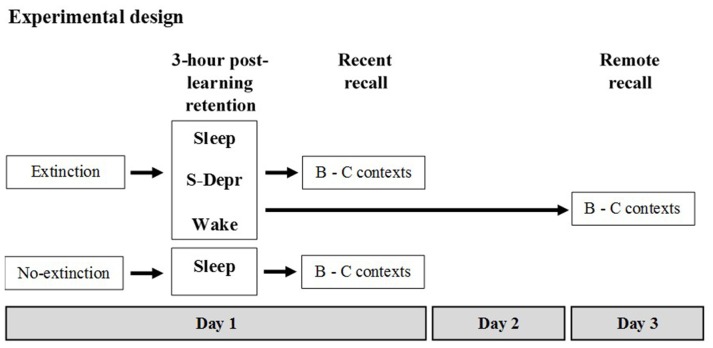
**Experimental design (see text for details).** After familiarization to the different contexts used in the experiment and after having acquired the operant behavior (rewarded lever press) on the day before, five groups of rats were subjected to Extinction training in Context B (Day 1). A 6th No-extinction control group remained awake in their home cages during this period. Extinction training was followed by a 3-h retention period during which rats of the Sleep groups slept, rats of the S-Depr groups were deprived from sleep, and the rats of the Wake group remained spontaneously awake. The post-learning retention period for the Sleep, S-Depr and No-extinction control groups took place between 10:00 a.m. and 1:00 p.m. of the light phase. For the Wake group the retention period took place between 10:00 p.m. and 01:00 a.m. of the dark cycle. Recall was tested in the extinction Context B and in a different Context C either right after the 3-h retention period (in the Sleep/Recent, the S-Depr/Recent, the Wake/Recent and the No-extinction control groups) or 48 h after Extinction training (Day 3, Sleep/Remote, S-Depr/Remote groups). Data from the S-Depr/Recent and Wake/Recent groups were pooled for analyses because these groups showed closely comparable performance at the recall test.

Memory for an extinguished operant behavior (food rewarded lever press) was tested in different groups of rats with the 3-h retention interval following extinction learning containing either morning sleep (Sleep, *n* = 16), morning sleep deprivation (S-Depr, *n* = 16), or spontaneous evening wakefulness (Wake, *n* = 16). Then, retrieval was tested (Recent recall). In two additional groups, a Sleep (*n* = 9) and S-Depr group (*n* = 11), recall was tested after 48 h (Remote recall). A sixth group (No-extinction control, *n* = 8) did not undergo extinction but was merely retested on the task at the Recent recall, as an estimate of the efficacy of extinction. Recall was tested either in the same context as during extinction learning (context B) or in a new (context C). For the Sleep and S-Depr groups as well as for the No-extinction control group all behavioral tests were performed during the light phase whereas for the Wake group tests were performed during the dark phase (for a summary of the design and procedures, see Figure 1).

Before the experiment proper started, rats in all groups were: (i) subjected to handling and food restriction regimes; (ii) pre-exposed to the different contexts (A, B, C) used in the experiments; and (iii) underwent lever press shaping procedures and the acquisition of the operant behavior (lever press response) in context A. The experiment proper started on Day 1 with extinction learning of the lever-press in context B, followed by a 3-h post-learning period containing either morning sleep, sleep deprivation or evening wakefulness. This was followed immediately (Recent) or with a delay of 48 h (Remote) by a recall test.

#### Handling and Food Restriction

During the 14 days before acquisition, the rats were handled for 4 min each day to habituate them to the experimental setting. Animals were gradually food restricted until they met the criterion of weighing 85% of their normal body weight before the experiment started. To achieve comparable food restriction during the test phase, Remote recall groups received 5–10 g/day during the retention interval between the extinction training and the Remote recall test.

#### Pre-Exposure to Contexts, Shaping of the Lever Press Response and Acquisition of Operant Response

Two days before the experiment proper, the rats were familiarized with the three different contexts (A, B, C) used in the experiments. To prevent any disrupting effects of context novelty, rats were exposed to each context for 15 min, according to standard procedures in extinction research (e.g., Bouton and Ricker, 1994). Note that context C, although it was pre-exposed to the rat *per se*, remains “novel” in terms of its genuine context function, i.e., serving as context for a specific learning history. The order of the context presentation was counterbalanced across rats in each group. During this phase the lever was removed from the chamber and the rat freely explored the contexts. Then, after a 10–30 min break, the magazine training and lever press shaping procedures were performed in context A. During the magazine training, ~12 pellets were manually delivered into the feeder in order to prime the animal to associate the feeder sound with the availability of food. Thereafter, the lever was introduced to the chamber and successive approximation to the lever press behavior was reinforced with one pellet until the animal got 90 pellets of reinforcement.

One day before the experiment proper, the rat acquired the operant response in context A. In this phase the animal learned to press the lever to get the reward. Rats were exposed to a continuous reinforcement schedule, i.e., each lever press was followed by one food pellet. The acquisition phase took 15 min.

#### Extinction Phase

The experiment proper started with the extinction learning phase, followed by the 3-h post-learning retention period and the Recent recall test. Extinction learning was done in context B and consisted of four 20-min blocks where the animal did not receive any reinforcement upon pressing the lever. The blocks were separated by 10-min breaks which the rat spent in its home cage in the same experimental room. Extinction was considered successful when the animal did not press the lever more than once per minute during the last 10 min of the 4th extinction block. If the animal did not reach this criterion, this last extinction block was extended to a maximum of 50 min. Rats of the No-extinction control group spent this phase awake in their home cage. As we aimed to test the acute effects of a single period of sleep, our extinction protocol deviated from standard protocols of extinction learning typically extending over several days (Todd, 2013). Thus, rather than a random reinforcement schedule we used a continuous reinforcement schedule during acquisition of the to-be-extinguished operant behavior, which is known to facilitate subsequent extinction (Ferster and Skinner, 1957). For this reason we also reduced extinction training to four 20-min blocks performed in a single session after the retention interval. As a consequence, using the 10-min interval of the last block to assess extinction (as well as using the final 3 min of training to assess prior acquisition of the operant behavior) was considered to most validly reflect the strength of the respective behaviors. Indeed, exploratory analyses using different interval duration for measuring extinction (and acquisition) revealed essentially the same results.

#### Post-Learning Retention Period

In the 3-h retention period following extinction learning, animals of the Sleep/Recent and Sleep/Remote groups (as well as the No-extinction control group) slept in their home cages, whereas the S-Depr/Recent and S-Depr/Remote groups were deprived from sleep during this period. Sleep deprivation was achieved by “gentle handling” to avoid stress (Hagewoud et al., 2010; Colavito et al., 2013). The procedure was initiated as soon as the animal showed signs of sleep and involved tapping on the cage, gently shaking the cage and, if necessary, disturbing the nest building behavior. The Wake/Recent group was spontaneously awake during this interval, with a few exceptions where the animal needed slight stimulation to stay awake. The post-learning retention period, for the Sleep, S-Depr and No-extinction control groups took place between 10:00 a.m. and 1:00 p.m. of the light phase, i.e., during their natural rest phase. For the Wake group the retention period took place between 10:00 p.m. and 01:00 a.m. of the dark cycle, i.e., during their natural active phase. Animals were videotaped during the post-learning retention period for offline scoring of sleep.

#### Recent and Remote Recall

In the Recent groups (i.e., the Sleep/Recent, the S-Depr/Recent and the Wake/Recent groups) recall was tested right after the 3-h post-learning retention period whereas in the Remote groups (i.e., the Sleep/Remote and the S-Depr/Remote groups) recall was tested 48 h later. For recall testing the rat was exposed for 10 min to the lever in the same context (B) as the extinction context and in a different context (C), with the order of contexts counterbalanced across rats. Recall tests in the different contexts were separated by a 15-min break which the rat spent in its home cage. During the recall test the animal did not receive reinforcement.

#### Apparatus

Acquisition and extinction of the lever press response took place in a standard operant chamber (32.7 cm × 27.5 cm; height: 23.8 cm). Two of the four side walls were made of polycarbonate, the other two were made of aluminum. One of the aluminum walls had a stainless steel food cup (diameter: 2 cm) centered in a wider 2 cm × 5 cm compartment (height 3 cm). The lever was located to the right of the food cup (3 cm × 5 cm). Food pellets (Noyes Precision Pellets PJFSC 0045, Research diets) were delivered by a food dispenser. All operant chamber events were recorded by ABET II Operant Chamber software (Model 89501, version 2.15). To differentiate the A, B and C contexts the floor, walls and orientation of the chamber in the room were changed. Context A was characterized by walls covered with a checkerboard pattern of 4 cm × 4 cm black/white squares, a smooth floor, dim white light, with the chamber turned counter-clockwise by 90° from a reference orientation in the experimental room. The characteristics of B and C contexts were counterbalanced across rats. One of these contexts featured walls covered by black and white horizontal lines, floor gratings, white light, with the chamber turned counter-clockwise by 45° from the reference orientation. The other context featured rugged transparent walls, red light, with the chamber turned counter-clockwise by 180°. The chamber was cleaned with 96% alcohol after each phase.

### Data Reduction and Statistical Analyses

Behavioral signs of sleep were determined offline from videotaped post-learning intervals using Camtasia Studio 8.0 video software (Techsmith, USA), with sleep identified whenever the rat displayed a typical sleep posture and stayed immobile for at least 5 s (Inostroza et al., 2013; Borquez et al., 2014). Operant behavior during acquisition, extinction learning and recall tests was expressed in lever presses per min. To test the contextual specificity of the extinction memory a discrimination index (DI) of the lever press response was calculated as follows: (Average response in C − Average response in B)/(Average response in C + Average response in B). A DI = 1 indicates maximal context specificity, i.e., extinction memory is present only in the original extinction context B, a DI = 0 indicates no context specificity.

Statistical analyses were based on analysis of variance (ANOVA) that, depending on the specific comparison, comprised a group factor representing the post-learning Retention conditions (Sleep/Recent, S-Depr/Recent, Wake/Recent, Sleep/Remote, S-Depr/Remote, No-extinction control), a repeated measures factor “Block” representing the four 20-min blocks of the extinction learning phase, and a repeated measures factor “Context” representing the different contexts (B, C) during recall testing. Significant global ANOVA main and interaction effects were followed by *post hoc t* test. One-sampled *t*-tests were performed to test whether the DI differed from zero, and independent sample *t* tests were performed to compare the DI between groups. Finally, Pearson’s correlation coefficients were calculated (for the Sleep/Recent and Sleep/Remote groups) between sleep time and recall performance in contexts B and C. For all analyses, SPSS software was used (IBM, Armonk, NY, USA).

## Results

### Acquisition and Extinction Learning

**Figure 2:**
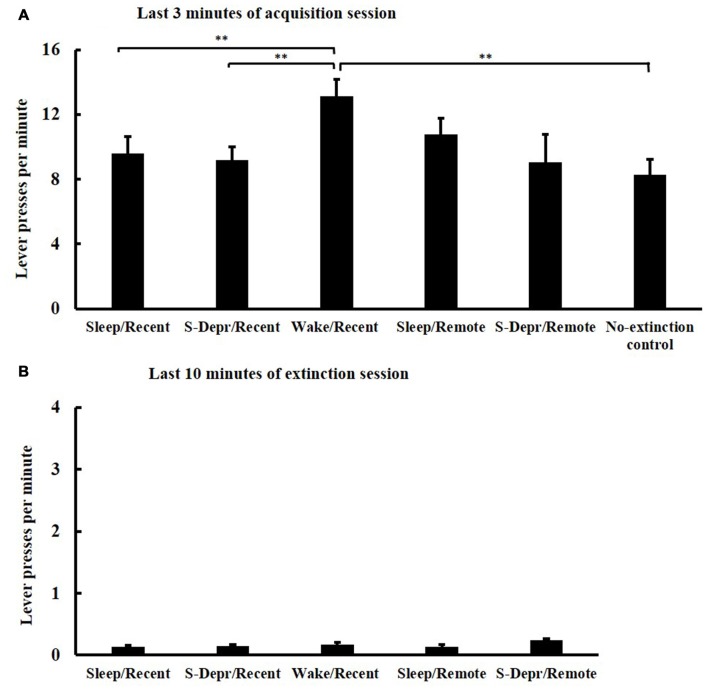
**Mean (±SEM) lever presses per minute (A)** at the last 3-min block of the acquisition session and **(B)** at the last 10 min of the extinction training, separately for the Sleep/Recent, S-Depr/Recent, Wake/Recent and Sleep/Remote, S-Depr/Remote and No-extinction control groups. The No-extinction control did not undergo extinction. Significance is indicated for *post hoc* pairwise comparisons, **p < 0.01.

All groups (Sleep/Recent, S-Depr/Recent, Wake/Recent, Sleep/Remote, S-Depr/Remote, No-extinction control) showed a comparable increase in lever press responses across the five 3-min blocks of the acquisition phase, indicating that all groups successfully acquired the operant behavior (*F*_(4,67)_ = 28.002, *p* > 0.001, for Block main effect). Performance during acquisition differed among groups (*F*_(4,63)_ = 2.274, *p* > 0.012, for Group main effect), such that the Wake/Recent group showed generally higher response rates than both the Sleep/Recent and S-Depr/Recent groups, possibly reflecting a circadian influence (*t*_(30)_ = −2.639, *p* < 0.05 and *t*_(30)_ = −2.973, *p* < 0.01, respectively). *Post hoc t*-tests confirmed that there were no significant differences in acquisition performance on the last 3-min block (except that in the Wake/Recent group response rates were higher than the other groups, *p* < 0.01, Figure 2A).

In all five groups, lever press responses diminished across the four blocks of the extinction phase (*F*_(3,61)_ = 51.209, *p* < 0.001, for Block main effect) indicating that all groups effectively extinguished the operant behavior. Extinction was comparable among the groups, with no significant difference in performance among groups on the last 10 min of the 4th block (*p* > 0.071; note, these analyses did not include the No-extinction control group, Figure 2B). Also, the length of the 4th extinction block, which varied depending on when the animal reached criterion performance, was comparable for all groups (29.85 ± 9.4 min, *p* > 0.11).

### Effects of Post-Learning Sleep on Recent and Remote Recall

To test whether sleep benefits consolidation of the operant extinction and to test changes in the context specificity of the extinction memory over time, we compared the rats’ performance at both Recent and Remote recall tests, for all post-learning conditions (Sleep, S-Depr, Wake) and for each Context (extinction context B and new context C). Because there were no differences in performance between the S-Depr/Recent and Wake/Recent groups at the Recent recall tests (all *p* > 0.779) these groups were pooled (and termed S-Depr/Recent). A global 2 (Recent/Remote) × 2 (Sleep/S-Depr) × 2 (Context B/Context C) repeated measure ANOVA revealed significance for the 3-way interaction (*F*_(1,64)_ = 7.138, *p* = 0.01), in addition to strong main effects for the Sleep/S-Depr factor (*F*_(1,64)_ = 15.440, *p* < 0.001) and the Context B/context C factor (*F*_(1,64)_ = 4.780, *p* < 0.05). The latter main effects indicated that the suppression of lever press responses was generally stronger for the Sleep than S-Depr groups and also stronger when tested in the extinction context B than in the novel context C.

**Figure 3:**
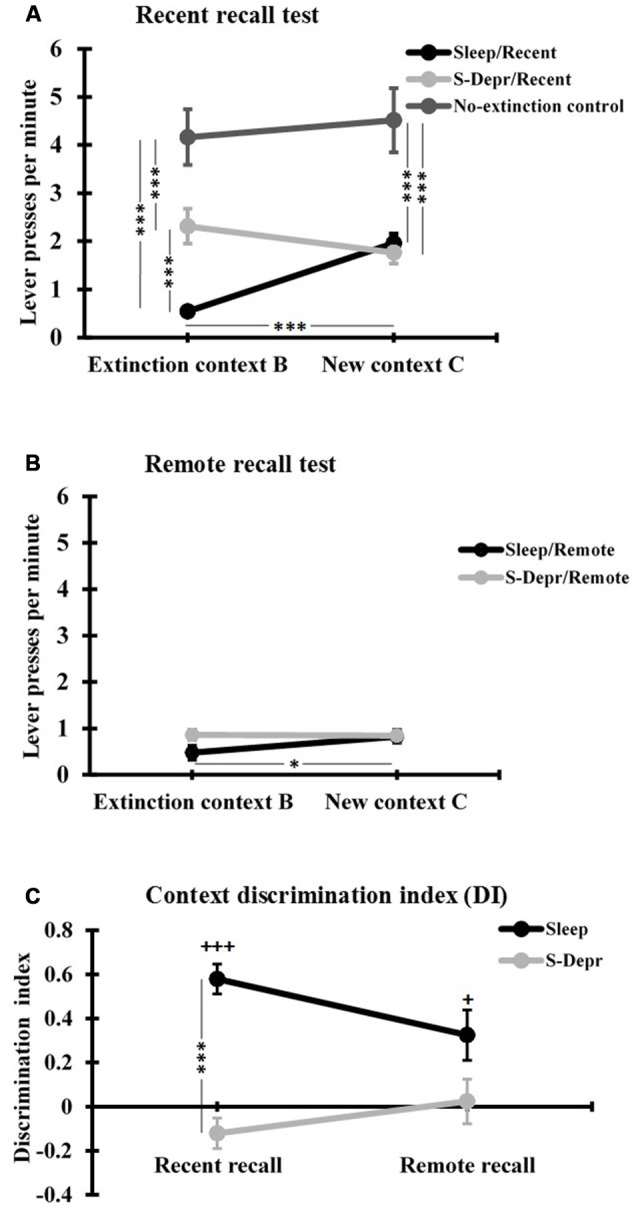
**(A)** Mean (±SEM) lever presses per minute at the Recent recall, for the Sleep/Recent, S-Depr/Recent and No-extinction control groups, and **(B)** at the Remote recall, for the Sleep/Remote and S-Depr/Remote groups, during testing in the extinction context (Context B) and in the different context (Context C). **(C)** Discrimination index (DI) for the lever press responses in context B and C of the Sleep and S-Depr groups at the Recent and Remote recall tests. Significance for *post hoc* pairwise tests is indicated ***p < 0.001, *p < 0.05. In **(C)** ^+++^*p* < 0.001, ^+^*p* < 0.05 indicate significance against “0”.

*Post hoc t*-tests conducted to clarify the 3-way interaction, revealed that at the Recent recall test the mean rate of lever presses/min in context B (extinction context) was significantly lower for the Sleep than S-Depr group (*t*_(46)_ = −4.396, *p* < 0.001, Figure 3A), whereas Recent recall performance in the novel context C did not differ between Sleep and S-Depr groups (*p* = 0.723). These data indicate that sleep during a 3-h retention period after extinction learning enhances memory for the learned extinction, however, only in the context in which extinction was learned. In fact, only in the Sleep group was suppression of the lever press response during Recent recall testing significantly stronger in the extinction context B than in the novel context C (*t*_(15)_ = −6.317, *p* < 0.001), indicating that this group showed a renewal of the response when tested in a novel context (Figure 3A). Consistent with this pattern, the DI as a measure of contextual specificity of extinction memory, was significantly higher in the Sleep/Recent than S-Depr/Recent group (*t*_(46)_ = −4.396, *p* < 0.001, Figure 3C). One-sampled *t*-tests revealed that only the Sleep/Recent group displayed a DI significantly above zero, confirming the context specificity of extinction memory formed after post-learning sleep (Sleep/Recent: *t*_(15)_ = 8.510, *p* < 0.001; S-Depr/Recent: *t*_(31)_ = −1.754, *p* = 0.089).

In contrast, at the Remote recall no significant differences were found between the Sleep/Remote and S-Depr/Remote groups or contexts, except that after sleep (i.e., the Sleep/Remote group) rats still showed slightly lower response rates in the original Context B than in Context C (*t*_(8)_ = −2.784, *p* = 0.024, Figure 3B). Indeed, response rates at Remote recall were rather low in all conditions and did not differ from that observed in the Sleep/Recent group in the original extinction context B (all *p* > 0.071). For the Sleep conditions, it appeared that the initially (i.e., at the Recent recall) context specific extinction became more general after 48 h. Accordingly, analysis of the DI indicated a reduction in context specificity from Recent to Remote recall which approached significance after sleep (*t*_(23)_ = 2.028, *p* < 0.054; Figure 3C), although the DI in the Sleep/Remote groups was still significantly greater than zero (*p* = 0.02). The pattern for the S-Depr conditions indicated a general enhancement in extinction from Recent to Remote recall, with no change in the context specificity of learned extinction. In fact, the DI did not change significantly from Recent to Remote recall after sleep deprivation (*p* = 0.277) and did also not significantly differ from zero in the two conditions (S-Depr/Recent: *p* = 0.143, S-Depr/Remote: *p* = 0.814). The difference in the DI between the Sleep/Remote and the S-Depr/Remote also failed to reach the 5%-level of significance (*t*_(18)_ = 1.977, *p* = 0.064), suggesting that with increased time after extinction learning the context specificity of extinction learning converges to zero independently of whether or not learning was followed by sleep.

The No-extinction control group which did not undergo extinction training but otherwise followed the same procedures as the Sleep/Recent group showed, as expected, distinctly enhanced rates of lever press responses in both contexts B and C (*F*_(2,53)_ = 19.99 and *F*_(2,53)_ = 13.875, *p* < 0.001, for main effects of Group in sub-ANOVA on the Sleep/Recent, S-Depr/Recent and No-extinction control group, Figure 3A), which did not differ between context B and C (*t*_(7)_ = −0.69, *p* = 0.510). This finding confirms the efficacy of extinction training and excludes substantial contributions of non-specific forgetting processes to the differential effects of sleep and sleep deprivation on context specific extinction memory.

The time spent asleep during the 3-h post-learning retention interval did not differ between groups (Sleep/Recent: 69.43 ± 17.13 min; Sleep/Remote 71.44 ± 12.56 min; *t*_(23)_ = −0.3, *p* = 0.762). Sleep time was not correlated with extinction memory performance in any of the conditions (Sleep/Recent in context B: *r* = −0.39, *p* > 0.330; Sleep/Recent in context C: *r* = −0.06, *p* > 0.817; Sleep/Remote in context B: *r* = −0.46, *p* > 0.209; Sleep/Remote in context C: *r* = 0.23, *p* > 0.546). There were also no significant differences between groups in the sleep onset (Sleep/Recent: 44.73 ± 34.87 min; Sleep/Remote 31.42 ± 11.97 min; *t*_(19)_ = 1.32, *p* = 0.202).

## Discussion

We examined the effects of post-learning sleep on memory for the extinction of an operant behavior (rewarded lever press response) and on the context specificity of this extinction memory in rats using a Recent (after 3-h) and a Remote recall test. The two main findings were: (i) At the Recent recall test, sleep compared with wakefulness enhanced the extinction memory but only if recall was tested in the same context (context B) as during extinction learning, indicating that 3 h of post-learning sleep enhances a context specific extinction memory; (ii) At the Remote recall test (48 h later), the Sleep group showed—in comparison with Recent recall testing—also improved recall of the extinction memory when tested in the novel context (context C), with this improvement reaching a level only slightly (but still significantly) lower than that for testing in the extinction context B. Thus, at remote testing the Sleep group still exhibited significant context discrimination (as is also indicated by the significance for the context DI in the Sleep/Remote group—Figure 3C). Interestingly, however, at the Remote recall test, extinction memory improved—in comparison with Recent recall testing—also after sleep deprivation in both contexts (B and C) such that recall performance was not anymore statistically different from that seen in the respective Sleep/Remote group. This pattern indicates an improvement and contextual generalization of extinction memory over 48 h after learning that is independent of the occurrence of sleep immediately after learning.

Comparing our findings in rats with those in other species like honey bees, mice and humans, involves the risk of premature over-generalization, as there are undoubtedly distinct differences in the systems mediating sleep, memory formation and extinction learning. Nevertheless, it is worthwhile mentioning that our results on the Recent recall test agree with evidence in honey bees, likewise showing that the formation of an extinction memory regarding an appetitive behavior is enhanced by sleep following extinction learning (Hussaini et al., 2009). It has even been speculated that in honey bees this sleep-dependent consolidation of extinction might follow a two-stage systems consolidation processes basically similar to that observed in rodents, with the antennal lobe and mushroom bodies, rather than the hippocampus and neocortex, as the principal structures supporting initial and long-term storage, respectively (Vorster and Born, 2015). Also in mice, extinction of cued fear conditioning was enhanced when sleep followed extinction learning (Melo and Ehrlich, 2016). This effect was independent of the circadian phase which agrees with the present results. Concurring with the present results, in that study the effect of sleep on fear extinction memory appeared to be context specific, as renewal effects with testing fear elicitation in a context different from the extinction context were robustly observed also in the post-learning sleep condition. In rats, post-learning sleep enhanced memory formation in an operant go/nogo conditional discrimination task (Borquez et al., 2014). Typically, in such tasks the rats are first trained on the “go” behavior (e.g., to press a lever to get reward) and then, not to express this behavior (“nogo”) once a discriminative stimulus is present (e.g., a light), with the nogo aspect of the task resembling extinction learning. Interestingly, testing immediately after the 80-min post-learning retention interval in that study (Borquez et al., 2014) revealed that sleep mainly benefitted the nogo response. However, contrasting with the present findings, the effect of sleep on the nogo memory was comparable if the recall test was conducted in the same context as in the learning context or in a different context. In combination with those observations, the present findings highlight the fact that extinction is a behavior strongly associated with the specific context in which it was acquired and that, contrasting with mere nogo learning, sleep after extinction learning also enhances the context specificity of this inhibitory behavior.

A focus of our study was the effect of sleep on the context specificity of extinction memory. The transformation of contextually detailed episodic memory into de-contextualized memories that can be flexibly used in quite different conditions is considered a gradual process (Inostroza and Born, 2013). This is why we, here, added a Remote recall (after 48 h) to the Recent recall condition. Our results indeed show a generalization of the extinction memory to the novel context, which was not used for extinction learning, at Remote testing. However, contrary to our expectation, this increased context generalization (i.e., decreased context specificity) occurred independently of whether the rat slept after extinction training or not. Thus, to generalize extinction memory, sleep does not need to occur immediately after post-learning sleep. However, sleep occurring during a later time window might still be necessary, considering also that the rats of our S-Depr/Remote group typically slept immediately after the 3-h period of sleep deprivation. Based on human experiments, it has recently been suggested that hippocampus-dependent and hippocampus-independent tasks may depend on two distinct processes of consolidation, each evolving through different dynamics during sleep (Schönauer et al., 2015). For hippocampal-dependent tasks post-learning sleep has an immediate enhancing effect (Prince et al., 2014). However, this enhancement is typically transient because the hippocampus, by serving as a buffer that intermediately stores the experienced information, enables consolidation of the information also during delayed sleep periods. As to the present findings, sleep during the 3-h post learning retention period enhanced extinction memory at the Recent recall, in comparison with post-learning wakefulness. This enhancement faded at the Remote recall because, here, rats of the S-Depr group had recovered sleep and this recovery sleep presumably effectively enhanced consolidation of the extinction memory also in this group. Whether sleep acting on hippocampus-dependent memories with some delay (i.e., after 3 h) also particularly favors the generalization and de-contextualization of the extinction memory—as observed here for the Sleep/Remote and S-Depr/Remote groups—cannot be answered based on the present experiments. An alternative explanation of this decrease in context specificity likewise seen at Remote testing in the Sleep and S-Depr groups, is that it simply originates with the passage of time (Wiltgen and Silva, 2007; Winocur et al., 2007), for example, as a consequence of forgetting of context that occurs independently of sleep (Cox et al., 2014; Migues et al., 2016).

Our study design excluded a number of factors that could have confounded the effect of sleep on extinction memory formation. As performance at the extinction and test sessions was closely comparable between the Wake and S-Depr groups (which were tested in the beginning of the rest and active phase, respectively), it is unlikely that fatigue or the circadian rhythm substantially contributed to the effects of sleep on recall performance. Moreover, the gentle handling procedure used to deprive rats from sleep reduced possible confounds of stress and associated corticosterone release to a minimum (Hagewoud et al., 2010; Melo and Ehrlich, 2016). Interestingly, we found a non-specific increase in lever press responses during acquisition of the operant response in the Wake group tested in the active phase compared with the other groups. This increase likely reflects a circadian effect on hunger and food intake (Panda et al., 2002), which would also explain why it did not occur during the extinction sessions of the experiment proper but only during acquisition of the operant behavior when the reward pellets were delivered.

Also, our No-extinction control assured an immediate effect of the experimental sleep manipulations on the formation of an extinction memory, rather than on a possible forgetting of the originally learned lever press response. This is the more important as in this study we achieved successful extinction learning in only a single session (preceding the experimental retention period) whereas other studies typically use repeated sessions over several days in order to effectively induce operant extinction (Rescorla, 2008; Bouton et al., 2011; Todd et al., 2012). Rates of lever presses in the No-extinction control group were in the extinction context (B) more than eight-fold higher than in the Sleep/Recent group which underwent extinction, and in the novel context C still two-fold higher than in the S-Depr/Recent group. The pattern strongly argues for an immediate impact of sleep on extinction memory rather than on forgetting of the original operant behavior, and makes such forgetting similarly unlikely as a factor explaining performance at the Remote recall, although this was not directly tested here.

Extinction learning might be in general a rather useful model for the study of active systems consolidation during sleep, and of putatively associated processes transforming memory into de-contextualized generalized representations in the hippocampus-dependent memory system which is more precisely termed a prefrontal-hippocampal memory system as it strongly involves the prefrontal cortex at encoding and retrieval (Preston and Eichenbaum, 2013; Eichenbaum, 2017). Extinction occurs in rather different learning paradigms, such as classical fear conditioning and appetitive operant conditioning which rely on distinct neural systems. Thus, acquisition of conditioned fear involves the amygdala and hippocampus (Orsini and Maren, 2012) whereas appetitive operant conditioning as well as its consolidation during sleep has been more closely linked to structures like the ventral striatum, amygdala, hippocampus and the medial prefrontal cortex (mPFC; Pennartz et al., 2004; Cardinal, 2006; Lansink et al., 2010). However, although quite different brain substrates are involved in the acquisition of these behaviors, extinction of these behaviors might share a critical involvement of the mPFC (Peters et al., 2009). In fear conditioning, the mPFC mediates the consolidation of extinction (Herry et al., 2010) while the hippocampus mediates the context-dependent expression of the fear extinction (Ji and Maren, 2007). In operant conditioning, the mPFC is involved in the contextual control of the extinction (Eddy et al., 2016; Wang et al., 2016) while the expression of extinction appears to be mediated via projections to the ventral striatum (Ghazizadeh et al., 2012). Whether sleep affects extinction learning in general, and to what extent such effects might conveyed via the mPFC is an open question, warranting research directly comparing the effects of sleep on the formation of different kinds of extinction memory.

Such research is all the more relevant as operant extinction, as tested here, is of utmost clinical relevance, e.g., in learning-based therapies of drug addiction where the lack of generalization of the extinguished addiction behavior from the clinical setting to the patient’s everyday life represents a major problem. Differing from our expectation, this study shows that post-learning sleep is not necessary for the gradual formation of a generalized extinction memory. However, our study also does not exclude such contribution, as the formation of a generalized extinction memory does not need to be necessarily linked to the occurrence of sleep immediately after learning. Thus, the central question arising from the present findings is why sleep immediately after learning strongly enhances context-specific extinction but, in the long run, this effect decays and the memory becomes better and less context-dependent even when the individual has been awake right after learning.

## Author Contributions

MB, MI, EV and JB designed the study. MPC, MI and JB wrote the manuscript. MPC and MB conducted the study, contributed equally; MPC and MI analyzed the data.

## Funding

This research was supported by a grant from the Deutsche Forschungsgemeinschaft (DFG) “Plasticity and Sleep”. MB received a scholarship from Comisión Nacional de Investigación Científica y Tecnológica (CONICYT), Chile.

## Conflict of Interest Statement

The authors declare that the research was conducted in the absence of any commercial or financial relationships that could be construed as a potential conflict of interest.
